# Healthy obesity and risk of accelerated functional decline and disability

**DOI:** 10.1038/ijo.2017.51

**Published:** 2017-03-14

**Authors:** J A Bell, S Sabia, A Singh-Manoux, M Hamer, M Kivimäki

**Affiliations:** 1 Department of Epidemiology and Public Health, University College London, London, UK; 2 MRC Integrative Epidemiology Unit at the University of Bristol, Bristol, UK; 3 INSERM, Centre for Research in Epidemiology and Population Health, Villejuif, France; 4 National Centre for Sport & Exercise Medicine, Loughborough University, Leicestershire, UK

## Abstract

**Background/Objectives:**

Some obese adults have a normal metabolic profile and are considered ‘healthy', but whether they experience faster ageing than healthy normal-weight adults is unknown. We compared decline in physical function, worsening of bodily pain and likelihood of future mobility limitation and disability between these groups.

**Subjects/Methods:**

This was a population-based observational study using repeated measures over 2 decades (Whitehall II cohort data). Normal-weight (body mass index (BMI) 18.5–24.9 kg m^−^^2^), overweight (25.0–29.9 kg m^−^^2^) and obese (⩾30.0 kg m^−2^) adults were considered metabolically healthy if they had 0 or 1 of 5 risk factors (hypertension, low high-density lipoprotein cholesterol, high triacylglycerol, high blood glucose and insulin resistance) in 1991/1994. Decline in physical function and worsening of bodily pain based on change in Short Form Health Survey items using eight repeated measures over 18.8 years (1991/1994–2012/2013) were compared between metabolic-BMI groups using linear mixed models. Odds of mobility limitation based on objective walking speed (slowest tertile) and of disability based on limitations in ⩾1 of 6 basic activities of daily living, each using three repeated measures over 8.3 years (2002/2004–2012/2013), were compared using logistic mixed models.

**Results:**

In multivariable-adjusted mixed models on up to 6635 adults (initial mean age 50 years; 70% male), healthy normal-weight adults experienced a decline in physical function of −3.68 (95% CI=−4.19, −3.16) score units per decade; healthy obese adults showed an additional −3.48 (−4.88, −2.08) units decline. Healthy normal-weight adults experienced a −0.49 (−1.11, 0.12) score unit worsening of bodily pain per decade; healthy obese adults had an additional −2.23 (−3.78, −0.69) units worsening. Healthy obesity versus healthy normal-weight conferred 3.39 (2.29, 5.02) times higher odds of mobility limitation and 3.75 (1.94, 7.24) times higher odds of disability.

**Conclusions:**

Our results suggest that obesity, even if metabolically healthy, accelerates age-related declines in functional ability and poses a threat to independence in older age.

## Introduction

Obesity is a considered a serious threat to public health.^1^ Health risks of obesity are largely mediated through disruptions to metabolism, which emerge in response to excess fat^2^ and which may subsequently lead to type 2 diabetes, cardiovascular diseases and premature mortality.^3, 4, 5^ As many as one-in-three obese adults at any given time, however, present without metabolic dysfunction in the form of metabolic risk factor clustering and are considered ‘healthy'.^6, 7^ This healthy subset was initially assumed to be protected from the adverse health consequences typical of obesity, but have since demonstrated strong tendencies to become insulin resistant,^8^ to progress to unhealthy obesity^9^ and to develop type 2 diabetes,^10^ and cardiovascular disease^11, 12, 13^ all at greater rates than normal-weight adults who are similarly healthy.

To our knowledge, excess risk for outcomes related to ageing among healthy obese adults has not been examined, although such evidence would form an important basis from which to advise on weight loss. Obesity is strongly linked with musculoskeletal impairments,^5, 14^ which often manifest clinically as osteoarthritis of the hip or knee,^15, 16^ one of the greatest and most enduring sources of pain, disability and diminished quality of life at older ages.^17, 18^ The presence of metabolic risk factors and high systemic inflammation may compound these adverse effects,^19, 20^ but given that the primary mechanism is thought to be mechanical strain placed on joints by excess fat,^14^ obesity with or without metabolic dysfunction may be hypothesised to limit functional ability to a similar degree. One study found that both healthy and unhealthy obese adults showed a higher likelihood of developing difficulties with walking or climbing stairs over a 7-year period than healthy normal-weight adults, suggesting worsened physical function in response to obesity itself.^21^ This finding has not been replicated and risk of other important age-related outcomes such as bodily pain and disability have not been compared between healthy obese and healthy normal-weight adults.

Using repeated measures over 2 decades in a well-characterised British cohort, the Whitehall II study, we aimed to compare long-term changes in two key indicators of functional ability—physical function and bodily pain—between middle-aged adults who were initially healthy obese and healthy normal-weight. We also compared the long-term risk of having a mobility limitation and of being disabled between these groups in order to examine potential for loss of independence.

## Materials and methods

### Study population

Longitudinal data were drawn from the Whitehall II cohort study, which recruited London-based men and women employed by the British government in 1985/1988.^22^ Questionnaire data are collected every 2–3 years, and clinical data are collected every 5 years. A combination of questionnaire data and clinical data from eight repeated assessments over 2 decades (baseline in 1991/1994; follow-up extending until 2012/2013) were used for present analyses. The University College London research ethics committee granted ethical approval for each phase of data collection. Participants provided written informed consent.

### Assessment of metabolic and obesity status

Data from a 1991/1994 clinical assessment were used to determine participants' initial obesity and metabolic status. Height and weight were measured objectively by a nurse and used to calculate body mass index (BMI) using the formula: weight (kg)/height (m)-squared. On the basis of World Health Organization BMI classifications,^23^ participants were considered either ‘normal-weight' (18.5–24.9 kg m^−^^2^), ‘overweight' (25.0–29.9 kg m^−^^2^), or ‘obese' (⩾30.0 kg m^−2^). Participants considered ‘underweight' (BMI <18.5 kg m^−2^) were excluded from analyses due to their rarity (*n*=72, 0.87% of the sample). On the basis of independent criteria,^6^ participants were also considered ‘healthy' if they had 0 or 1 of the following 5 metabolic risk factors: high-density lipoprotein (HDL) cholesterol <1.03 mmol l^−1^ for men and <1.29 mmol l^−1^ for women or use of lipid lowering medication; blood pressure ⩾130/85 mm Hg or use of anti-hypertension medication; fasting plasma glucose ⩾5.6 mmol l^−1^ or use of anti-diabetic medication; triacylglycerol⩾ 1.7 mmol l^−1^; homeostatic model assessment (HOMA) of insulin-resistance (fasting glucose × fasting insulin/22.5) >3.20 (90th-percentile value in 1991/1994).

### Assessment of physical function and bodily pain

Participants were asked to answer a series of 36 question items covering several domains of general health from the Short Form Health Survey (SF-36) at the time of metabolic and obesity status assessment (1991/1994) and at seven subsequent occasions (in 1995/1996, 1997/1999, 2001, 2002/2004, 2006, 2007/2009 and 2012/2013). Domains assessed by the SF-36 have been shown to be valid measures of overall health status in the general population^24^ and of change in overall health status in the Whitehall II cohort.^25^

Assessment of physical function was based on a sub-domain comprised of 10 items from the SF-36, which pertained to physical function over the past 4 weeks. Participants reported whether they considered their health to limit basic tasks, including vigorous activities (that is, running), moderate activities (that is, housework), lifting or carrying groceries, climbing several flights of stairs, or movements, which involve bending, kneeling and stooping. Response options for each item ranged from ‘not limited at all' to ‘limited a lot'.

The assessment of bodily pain was based on another sub-domain comprised of two items from the SF-36, which pertained to perceptions of bodily pain during the past 4 weeks, which asked participants to report how much bodily pain they experienced (response options ranging from ‘none' to ‘very severe') and how much this pain interfered with their normal work inside and outside of the home (response options ranging from ‘not at all' to ‘extremely').

Responses on each sub-domain were summed and scaled from 0 to 100 based on standard procedures for the SF-36,^26^ with higher scores representing better function/less bodily pain. Summary scores for each of physical function and bodily pain at all eight measurement occasions were used to estimate change over time, with decreasing scores indicating worsened physical function/bodily pain.

### Assessment of mobility limitation and disability

Mobility limitation was assessed on three occasions after assessment of metabolic and obesity status (in 2002/2004, 2007/2009 and 2012/2013). On each occasion, participants undertook a test of walking speed based on standard protocol,^27^ for which they completed a timed walk at their usual walking pace over a distance of 8 feet while wearing low-heeled closefitting footwear or while barefoot. Timing commenced once their foot hit the floor across the starting line, and stopped once their foot hit the floor after the end of the walking course. The test was repeated three times and the mean performance time of these three measurements was used for present analyses, measured in seconds (s). On the basis of established links with morbidity and mortality,^27, 28, 29, 30^ participants were considered to have a mobility limitation on each occasion if they were in the slowest (versus the intermediate/fastest) tertile of walking speed.

Disability was also assessed on three occasions after assessment of metabolic and obesity status (in 2006, 2007/2008 and 2012/2013). On each occasion, participants reported via questionnaire whether they considered themselves to have difficulty with any of 6 basic activities of daily living^31^ (dressing, walking across a room, bathing/showering, eating, getting in/out of bed and using the toilet). Participants were considered ‘disabled,' if they reported⩾1 (versus 0) limitation in any activity.

### Assessment of covariates

Covariates were assessed via questionnaire at the same time as metabolic and obesity status in 1991/1994. Participant age, sex and ethnicity (‘white' or ‘non-white') were recorded in addition to social status based on occupational position in the British government (‘administrative', ‘professional/executive', or ‘clerical/support'). Assessment of health behaviours included cigarette smoking status (‘never smoker', ‘ex-smoker' or ‘current smoker'), alcohol consumption in the previous week (‘abstainer' based on 0 units per week, ‘moderate drinker' based on 1–14 units per week for women and 1–21 units per week for men, or ‘high drinker' based on >14 units per week for women and >21 units per week for men), frequency of fruit and vegetable consumption (‘less than daily or daily', or ‘twice or more per day') and physical activity that was assessed by self-reported duration (hours per week) in activities of a moderate or vigorous intensity.

### Statistical analyses

Linear mixed models were used to compare mean change in physical function and bodily pain scores over eight measurement occasions, along with 95% confidence intervals (CI), by initial metabolic and obesity status in 1991/1994, each compared with the healthy normal-weight group. These models minimise selection bias from missing data by using data from all available follow-up occasions while accounting for differences in duration of follow-up and the correlated nature of repeated measures taken from the same individuals over time.^32^ Follow-up duration was used as the time variable, divided by 10 so that regression coefficients represent effects for change over 10 years. A random intercept and a random slope were fitted to allow individual differences in initial physical function/bodily pain score and change in these scores over time. Absolute change in each score was also calculated for each metabolic and obesity group based on intercept values taken at the reference groups of categorical covariates (for men; white ethnicity; administrative/highest occupational position; never smokers; moderate drinkers; at least twice-daily consumers of fruits and vegetables) and age centred on the sample mean (50 years). Predictors in the first model included metabolic and BMI status combination (6 groups), time, age, sex and ethnicity, each with time interactions fitted where significant. Predictors in the second model considered those of the first in addition to occupational position, smoking, alcohol, fruit and vegetable consumption, and moderate-to-vigorous physical activity, each with time interactions where significant.

Logistic mixed models were used to compare odds of having a mobility limitation and of having a disability between metabolic and BMI combination groups, each compared with healthy normal-weight. These models minimise selection bias due to missing data for the same reasons as mentioned for linear mixed models.^32^ Duration of follow-up was again used as the time variable with time expressed per 5 years instead of per 10 years due to shortened follow-up. The same 2-stage model adjustment strategy was otherwise applied as prior.

As some ethnic heterogeneity existed in the sample yet precise ethnic labels were not available for ascribing ethnic-specific BMI categories, analyses were repeated after excluding the 9% of participants who were of a non-white ethnicity. Analyses of change in physical function and bodily pain were also repeated after excluding those participants with only 1 available measure out of 8 on each outcome.

## Results

### Selection and characteristics of the study population

The Whitehall II cohort originally consisted of 10 308 participants recruited in 1985/1988.^22^ Of this original sample, 6641 participants (64.4%) had complete data on height and weight for the assessment of BMI and on each of five metabolic risk factors of interest as measured in the 1991/1994 clinical examination. Of these, six participants were excluded due to missing data on each of eight follow-up measures of physical function or bodily pain. All remaining participants had data on basic covariates for initial adjustments (age, sex and ethnicity). We excluded a further 392 participants from models adjusted for occupational position and health behaviours due to missing data on these covariates. Sample attenuation patterns were similar for outcomes of mobility limitation and disability, with the exception of a larger reduction (1306 participants) from the 6641 with BMI-metabolic data due to missing data on either outcome; data collection for these began later than for physical function and bodily pain.

Compared with participants who had metabolic-BMI data (the initial prerequisite for inclusion) and also had data on mobility (*n*=5507), those who had metabolic-BMI data but had missing data on mobility (*n*=1134) were older (51.1 vs 49.2 years, *P*<0.001), more likely to be female (34.7 vs 28.2%, *P*<0.001), more likely to be of a non-white ethnicity (13.3 vs 8.5%, *P*<0.001) and more likely to be of the lowest occupational position (27.1 vs 13.3%, *P*<0.001). Those with missing mobility data also had a higher smoking prevalence (21.2 vs 11.8%, *P*<0.001) and a higher likelihood of consuming fruit and vegetables less than daily (46.5 vs 37.4%, *P*<0.001), but were no less likely to consume high amounts of alcohol (14.1 vs 15.7%, *P*=0.168) or to be less physically active (3.4 vs 3.6 h/week, *P*=0.104). Participants with missing mobility data showed a higher prevalence of obesity (12.6 vs 9.4%, *P*=0.001) and of metabolic risk factor clustering (39.4 vs 32.6%, *P*<0.001). These comparison estimates were nearly identical among participants with vs without missing data on disability (Supplementary Appendix).

In total, up to 6635 participants contributed data for analyses, with the working sample size varying due to the nature of mixed modelling. Age of participants ranged from 39 to 63 years at the baseline assessment (mean 49.5 years) and 70.1% were men. Of the 3339 adults who were normal-weight, 80.5% were considered metabolically healthy; this proportion decreased with increasing BMI group: 56.3% of 2634 overweight adults were healthy and 34.0% of 662 obese adults were healthy. Further characteristics of participants who had complete data on metabolic and obesity status in 1991/1994 and at least 1 measure of physical function and bodily pain are shown in Table 1. Of those who had physical function and bodily pain scores at baseline, those who were healthy obese had lower (more adverse) scores than healthy normal-weight adults, these differences being substantial at 83.3 vs 92.1 for physical function, and 77.2 vs 83.0 for pain (both *P*<0.05).

### Change in physical function and bodily pain

**Figure 1:**
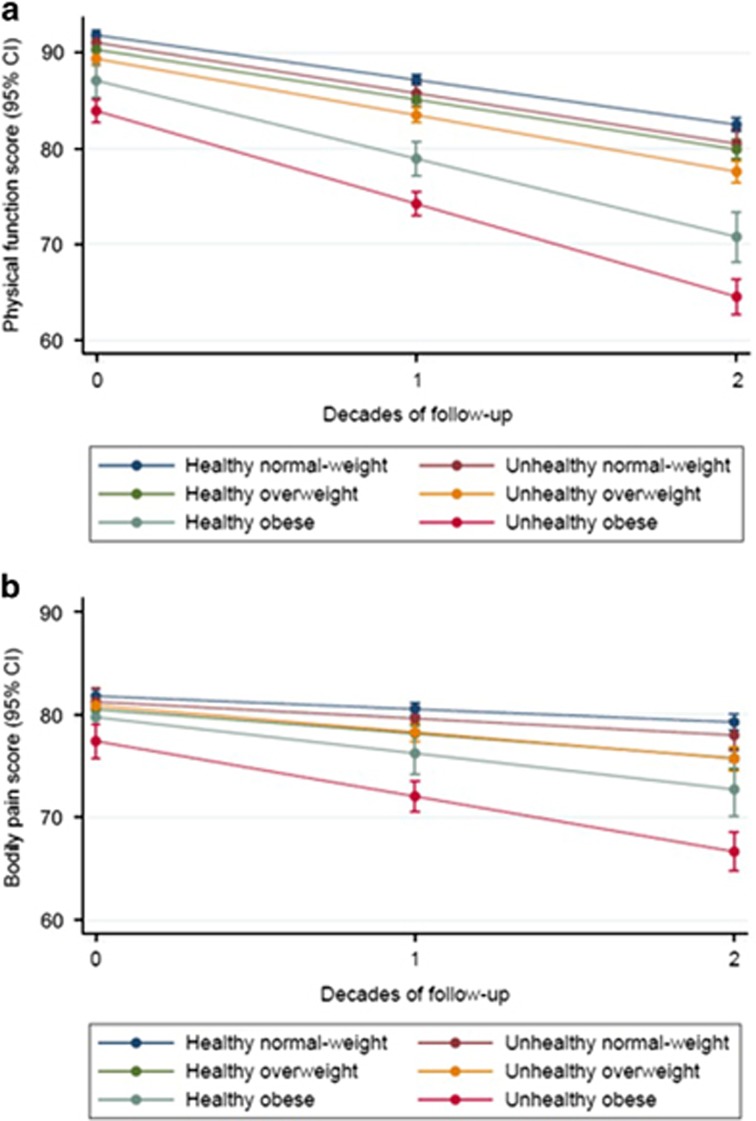
Decline in physical function (**a**) and worsening of bodily pain (**b**) over 2 decades by initial metabolic and obesity status. Models include adjustment for 1991/1994 values of age, sex, ethnicity, occupational position, moderate-to-vigorous physical activity, smoking, alcohol, and fruit and vegetable consumption.

Nearly all participants (*n*=6537; 98.5%) had data on at least two of eight measures for physical function, from which to base estimates of change (3707 participants, 55.9%, had data on all eight measures). The interaction term between sex, metabolic-BMI group and time in relation to physical function was not significant (*P*=0.925), indicating similar changes in physical function by metabolic-BMI group in both men and women. Over a mean follow-up of 18.8 years, decline in physical function score was seen among all metabolic and BMI combination groups over the follow-up period (Table 2). When adjusting for basic demographic factors, the healthy obese showed an additional −3.42 (95% CI=−4.80, −2.03) units decline per 10 years in physical function score than healthy normal-weight adults; this difference remained after additional adjustment for occupational position and health behaviours (−3.48, 95% CI=−4.88, −2.08 units; Figure 1). This decline was nearly two-times greater than among healthy normal-weight adults ((3.68+3.48)/3.68=1.95). The greatest decline was seen among unhealthy obese adults (additional −5.02, 95% CI=−6.06, −3.98 units) compared with healthy normal-weight adults, but this was not significantly greater than for healthy obese adults (*P*=0.068). Non-significant interaction terms of time with sex, alcohol consumption, physical activity, and fruit and vegetable consumption were removed from these models.

Again, nearly all participants (*n*=6538; 98.5%) had data on at least two of eight measures for bodily pain, from which to base estimates of change (3699 participants, 55.8%, had data on all eight measures). No strong evidence for an interaction between sex, metabolic-BMI group and time in relation to bodily pain was observed (*P*=0.054). A worsening of bodily pain score was also seen among all metabolic and obesity groups over follow-up (Table 2). This worsening was greater among healthy obese compared with healthy normal-weight adults when considering basic demographics (difference in 10-year change=−2.15, 95% CI=−3.66, −0.63 units); this difference remained after additional adjustment for social and behavioural factors (−2.23, 95% CI=−3.78, −0.69 units; Figure 1), equating to nearly a six-times greater worsening than that of healthy normal-weight adults ((0.49+2.23))/0.49=5.55). The greatest worsening was seen among unhealthy obese adults (difference in 10-year change=−4.10, 95% CI=−5.24, −2.95 compared with healthy normal-weight); there was weak evidence of this being greater than for the healthy obese (*P*=0.045). A non-significant interaction term of time with physical activity was removed from these models.

### Odds of mobility limitation and disability

**Figure 2:**
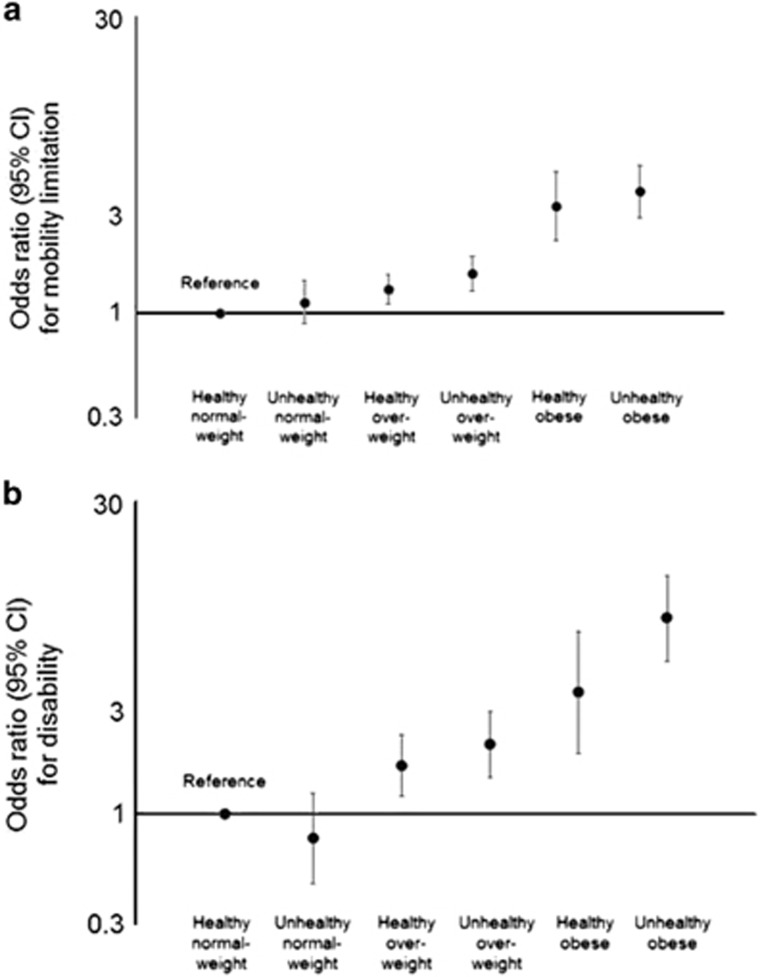
Likelihood of having a mobility limitation (**a**) and of having a disability (**b**) over 8.3 years by initial metabolic and obesity status. Models include adjustment for 1991/1994 values of age, sex, ethnicity, occupational position, moderate-to-vigorous physical activity, smoking, alcohol, and fruit and vegetable consumption.

Among 6641 participants whose metabolic and BMI status was assessed in 1991/1994, up to 5507 (82.9%) had at least one assessment of mobility limitation over a mean observation period of 8.3 years (3841 participants (57.8%) had all three assessments). The proportion of adults who had a mobility limitation over follow-up was lowest among healthy normal-weight adults at 29.1%, and highest among healthy obese and unhealthy obese adults, at 60.1 and 56.7%, respectively. Differences in odds of mobility limitation by metabolic and obesity group did not differ over follow-up (p for interaction of metabolic and BMI combination with time=0.36) and so this time interaction was removed; likewise for sex and ethnicity (*P*-values for interaction with time=0.099 and 0.175, respectively). Compared with healthy normal-weight adults, healthy obese adults showed 3.92 (95% CI=2.64, 5.80) times higher odds of having a mobility limitation over follow-up when adjusting for demographics; odds remained 3.39 (95% CI=2.29, 5.02) times higher when additionally adjusting for social and behavioural factors (Figure 2; Table 3). Raised odds of mobility limitation were highest among unhealthy obese adults at 4.01 (95% CI=2.98, 5.40) times higher than healthy normal-weight adults, however, this was not significantly higher than the healthy obese (*P*=0.48).

Among 6641 participants whose metabolic and BMI status was assessed in 1991/1994, up to 5616 (84.6%) had at least one assessment of disability over a mean observation period of 5.6 years (4434 participants (66.8%) had all three assessments). The proportion of adults who had a disability over follow-up was lowest among healthy normal-weight adults at 9.1%, and progressively higher among healthy obese and unhealthy obese adults at 18.6 and 27.0%, respectively. Again, a non-significant interaction of metabolic and BMI combination with time (*P*=0.34) provided no evidence that differences in odds of disability by metabolic and obesity group changed over follow-up, this time interaction was therefore removed; likewise for all other covariates except for age, which reached significance (*P*-value for interaction with time <0.001). Compared with healthy normal-weight adults, healthy obese adults were 3.84 (95% CI=2.01, 7.34) times more likely to be disabled when adjusting for basic demographic factors; these odds remaining elevated at 3.75 (95% CI=1.94, 7.24) times higher when additionally adjusting for social and behavioural factors (Figure 2; Table 3). The highest raised odds were seen among unhealthy obese adults (OR=8.37, 95% CI=5.25, 13.35 vs healthy normal-weight), there was some evidence of this being higher than for healthy obese adults (*P*=0.03).

### Sensitivity analyses

Results of sensitivity analyses are provided in Supplementary Appendix. Results of analyses that excluded the 9% of participants who were of a non-white ethnicity were largely unchanged; as were results of analyses of change in physical function and bodily pain that excluded participants with only 1 measurement of each outcome. A larger participant drop-out was observed for mobility limitation and disability than for physical function and bodily pain; a comparison of characteristics between included versus excluded participants for these former outcomes is given in Supplementary Appendix.

## Discussion

This study of 6635 men and women examined whether obese adults who are metabolically healthy experience faster ageing than normal-weight adults who are similarly healthy by way of greater declines in physical function, greater worsening of bodily pain, and higher likelihoods of having a mobility limitation and disability in older age. Our results showed that over the course of 2 decades, decline in physical function and worsening of bodily pain among initially healthy obese adults was two- and six-times greater than among initially healthy normal-weight adults, respectively. These changes occurred at similar rates for both healthy and unhealthy obese adults. A comparably higher likelihood of having a mobility limitation and of being disabled was also observed. This suggests that obesity, even if metabolically healthy, accelerates age-related declines in functional ability and poses a threat to independence in older age.

Comparisons of walking speed between healthy obese and healthy normal-weight groups is novel; only 1 previous study of women found that the healthy obese performed better than the unhealthy obese on a timed test of walking distance, but comparisons were not made with the healthy normal-weight.^33^ That study was also limited by a small sample size (total *n*=86) and a single measurement occasion; the present study considered three measurement occasions of walking speed spanning nearly a decade to provide a better estimate of usual walking capacity.

The likelihood of being disabled was somewhat lower among healthy obese than among unhealthy obese adults, but the difference between these two groups was small and not likely significant in terms of disability burden. Indeed, healthy obese adults are known to have a strong tendency to progress to an unhealthy obese state; this proportion is about one-half in the Whitehall II cohort after 20 years.^9^ Importantly, these progressions to unhealthy obesity occur at greater rates among adults who are initially healthy obese than among adults who are either healthy or unhealthy non-obese, likely reflecting causal effects of higher BMI on metabolic dysfunction and of higher BMI on lower physical activity as supported by Mendelian randomisation studies.^23, 34, 35^

Similar to previous studies, healthy obesity was defined here using an array of metabolic risk factors that are commonly measured in clinical settings, and such classifications based on the binary presence or absence of blood-based risk factors using cut-points may offer clinical relevance at the expense of scientific precision. Indeed, descriptive characteristics of participants at first measurement showed that healthy obese adults had more adverse levels of most metabolic risk factors than healthy normal-weight adults despite both groups being classified as ‘healthy' this is commonly observed across studies in this area. We did not analyse the already established associations of healthy obesity with metabolic decline,^9^ type 2 diabetes,^10^ cardiovascular disease^13^ or other chronic diseases^36^ as these are expected to mediate and not confound associations with functional outcomes. We considered only those activities of daily living which were considered basic and not instrumental in assessing disability because basic activities are thought to be more closely related to functional status and are more severe and limiting, whereas instrumental activities such as one's ability to manage money often relate more to cognitive functioning and can more readily be adapted to with informal caregiving.

### Strengths and limitations

Change in two key indicators of functional status were examined utilising up to 8 repeated measures over a follow-up period spanning 2 decades, providing a more comprehensive view of long-term change than previously possible. Mixed modelling was performed to make maximum use of all available data over the long follow-up period and to minimise the effects that selection bias due to missing data can have on results. The extent of missing data was largest for mobility and disability outcomes, with participants missing on these outcomes appearing more socioeconomically disadvantaged and less behaviourally and physically healthy than those with complete data; however, the impact of this selection bias is expected to be more modest here given the use of repeated measures on outcomes compared to what would be expected if a more restrictive sample was used for complete case analyses. The indicators of physical function and bodily pain used were also based on self-reported questionnaire items which are subject to biases in reporting and individual subjectivity; however, both objective and self-reported measures were used to assess functional limitations in the form of mobility limitation and disability, allowing for internal validation of self-reported findings and improved consistency of results.

## Conclusions

Our results suggest that obesity, even if metabolically healthy, accelerates age-related declines in functional ability and poses a threat to independence in older age. Long-term decline in physical function was nearly two-times greater, and worsening of bodily pain nearly six-times greater, among obese adults who are metabolically healthy than among normal-weight adults who are similarly healthy. The likelihood of developing a mobility limitation and of becoming disabled was also nearly four-times greater among healthy obese than among healthy normal-weight adults. Weight loss is therefore still advisable for healthy obese adults for the purpose of preserving the quality of later life.

## Figures and Tables

**Table 1:** Characteristics of participants in 1991/1994 by metabolic and obesity status in the Whitehall II cohort study (*n*=6635)

	*Healthy normal-weight (*n*=2688)*	*Unhealthy normal-weight (*n*=651)*	*Healthy overweight (*n*=1482)*	*Unhealthy overweight (*n*=1152)*	*Healthy obese (*n*=225)*	*Unhealthy obese (*n*=437)*
Female—*n* (%)	863 (32.1)	89 (13.7)^a^	481 (32.5)	193 (16.8)^a^	148 (65.8)^a^	172 (39.4)^a^
Age, years—mean (s.d.)	48.7 (6.0)	50.2 (6.0)^a^	49.5 (5.9)^a^	50.8 (6.0)^a^	49.7 (5.8)^a^	50.3 (5.9)^a^
Non-white ethnicity—*n* (%)	185 (6.9)	78 (12.0)^a^	139 (9.4)^a^	126 (10.9)^a^	42 (18.7)^a^	48 (11.0)^a^
Lowest occupational position—*n* (%)	357 (13.3)	73 (11.2)	268 (18.1)^a^	168 (14.7)	64 (28.7)^a^	104 (24.1)^a^
Consumes fruit and vegetables<daily—*n* (%)	947 (35.2)	264 (40.6)^a^	584 (39.4)^a^	512 (44.4)^a^	77 (34.2)	198 (45.3)^a^
Current smoker—*n* (%)	320 (12.6)	93 (14.9)	183 (13.2)	154 (14.2)	31 (14.6)	56 (13.7)
High alcohol consumption in previous week—*n* (%)	353 (13.2)	111 (17.1)^a^	236 (16.0)^a^	222 (19.4)^a^	31 (14.0)	68 (15.8)
Moderate-to-vigorous physical activity, h per week—mean (s.d.)	3.7 (4.1)	3.5 (3.9)	3.6 (4.2)	3.6 (4.0)	2.7 (3.1)^a^	2.7 (3.2)^a^
Systolic blood pressure, mm Hg—mean (s.d.)	115.9 (12.0)	127.5 (14.6)^a^	118.7 (11.2)^a^	128.0 (13.0)^a^	121.0 (13.5)^a^	130.6 (12.7)^a^
Diastolic blood pressure, mm Hg—mean (s.d.)	76.2 (8.4)	83.8 (8.9)^a^	79.0 (8.1)^a^	85.6 (8.6)^a^	80.6 (9.4)^a^	87.1 (8.9)^a^
Fasting glucose, mmol l^−1^—mean (s.d.)	5.1 (0.4)	5.6 (0.9)^a^	5.1 (0.4)	5.6 (0.8)^a^	5.0 (0.4)	5.7 (1.3)^a^
HOMA insulin resistance—mean (s.d.)	1.0 (0.8)	1.8 (1.3)^a^	1.4 (0.8)^a^	2.5 (2.1)^a^	1.7 (1.0)^a^	4.1 (4.5)^a^
Triacylglycerol, mmol l^−1^—mean (s.d.)	1.0 (0.4)	2.0 (1.2)^a^	1.2 (0.5)^a^	2.2 (1.2)^a^	1.2 (0.5)^a^	2.3 (1.2)^a^
HDL cholesterol, mmol l^−1^—mean (s.d.)	1.6 (0.4)	1.2 (0.4)^a^	1.5 (0.3)^a^	1.2 (0.3)^a^	1.5 (0.3)^a^	1.2 (0.3)^a^
Body mass index, kg m^−^^2^—mean (s.d.)	22.6 (1.6)	23.4 (1.3)^a^	26.7 (1.3)^a^	27.2 (1.4)^a^	32.4 (2.5)^a^	33.4 (3.4)^a^
Initial physical function score^b^—mean (s.d.)	92.1 (12.1)	90.9 (13.1)	89.6 (14.6)^a^	89.0 (14.1)^a^	83.3 (17.9)^a^	81.5 (18.4)^a^
Initial bodily pain score^b^—mean (s.d.)	83.0 (19.0)	83.3 (18.5)	81.2 (20.2)^a^	82.4 (19.4)	77.2 (21.8)^a^	77.5 (22.5)^a^

Participants described are those with the data on metabolic and obesity status and at least one measurement of physical function and bodily pain.

a Different from healthy normal-weight (*P*<0.05).

b On the basis of participants with a physical function and pain score in 1991/94.

**Table 2:** Decline in physical function and worsening of bodily pain per decade by initial metabolic and obesity status in the Whitehall II cohort study

	*Model 1*	*Model 2*
	B *(95% CI)*	B *(95% CI)*
*Decline in physical function per 10 years*^a^		
Decline in healthy normal-weight	−4.27 (−4.68, −3.86)	−3.68 (−4.19, −3.16)
Healthy normal-weight (*n*=2569)	0.00 (reference)	0.00 (reference)
Unhealthy normal-weight (*n*=615)	−0.74 (−1.60, 0.12)	−0.61 (−1.47, 0.26)
Healthy overweight (*n*=1420)	−0.68 (−1.30, −0.06)	−0.54 (−1.18, 0.09)
Unhealthy overweight (*n*=1070)	−1.48 (−2.17, −0.78)	−1.22 (−1.92, −0.52)
Healthy obese (*n*=205)	−3.42 (−4.80, −2.03)	−3.48 (−4.88, −2.08)
Unhealthy obese (*n*=401)	−5.18 (−6.20, −4.17)	−5.02 (−6.06, −3.98)

*Worsening of bodily pain per 10 years*^a^		
Worsening in healthy normal-weight	−1.15 (−1.60, −0.71)	−0.49 (−1.11, 0.12)
Healthy normal-weight (*n*=2560)	0.00 (reference)	0.00 (reference)
Unhealthy normal-weight (*n*=616)	−0.54 (−1.48, 0.39)	−0.36 (−1.31, 0.60)
Healthy overweight (*n*=1412)	−1.23 (−1.91, −0.56)	−1.10 (−1.80, −0.41)
Unhealthy overweight (*n*=1070)	−1.55 (−2.30, −0.79)	−1.31 (−2.09, −0.53)
Healthy obese (*n*=208)	−2.15 (−3.66, −0.63)	−2.23 (−3.78, −0.69)
Unhealthy obese (*n*=403)	−4.35 (−5.46, −3.24)	−4.10 (−5.24, −2.95)

Model 1 adjusted for age, sex and ethnicity in 1991/1994. Model 2 additionally adjusted for occupational position, moderate-to-vigorous physical activity, smoking, alcohol, and fruit and vegetable consumption in 1991/1994. Reference group for intercept is men in these analyses; interaction terms with sex were non-significant and findings were similar when analyses were repeated with women as the reference (Supplementary Appendix).

a Lower scores indicate worsened function/pain.

**Table 3:** Odds of mobility limitation and disability among adults over 8.3 years by initial metabolic and obesity status in the Whitehall II cohort study

	*Model 1*	*Model 2*
	*Odds ratio (95% CI)*	*Odds ratio (95% CI)*
*Odds of having a mobility limitation*^a^		
Healthy normal-weight (*n*=2023)	1.00 (reference)	1.00 (reference)
Unhealthy normal-weight (*n*=448)	1.22 (0.95, 1.56)	1.13 (0.88, 1.45)
Healthy overweight (*n*=1101)	1.44 (1.21, 1.71)	1.31 (1.10, 1.56)
Unhealthy overweight (*n*=812)	1.85 (1.52, 2.25)	1.57 (1.28, 1.91)
Healthy obese (*n*=148)	3.92 (2.64, 5.80)	3.39 (2.29, 5.02)
Unhealthy obese (*n*=275)	4.58 (3.41, 6.13)	4.01 (2.98, 5.40)

*Odds of having a disability*^b^		
Healthy normal-weight (*n*=2250)	1.00 (reference)	1.00 (reference)
Unhealthy normal-weight (*n*=502)	0.83 (0.51, 1.33)	0.77 (0.47, 1.25)
Healthy overweight (*n*=1208)	1.72 (1.25, 2.36)	1.70 (1.22, 2.36)
Unhealthy overweight (*n*=901)	2.22 (1.57, 3.14)	2.13 (1.49, 3.04)
Healthy obese (*n*=161)	3.84 (2.01, 7.34)	3.75 (1.94, 7.24)
Unhealthy obese (*n*=333)	8.89 (5.64, 14.00)	8.37 (5.25, 13.35)

Model 1 adjusted for age, sex and ethnicity in 1991/1994. Model 2 additionally adjusted for occupational position, moderate-to-vigorous physical activity, smoking, alcohol, and fruit and vegetable consumption in 1991/1994.

a Mobility limitation defined as being in the slowest vs fastest/intermediate tertile of walking speed.

b Disabled defined as having⩾1 out of 6 limitations in basic activities of daily living.
