# Supplementary material for: Healthy obesity and risk of accelerated functional decline and disability
Source: Int J Obes (Lond). 2017 Mar 14;41(6):866–72. doi: 10.1038/ijo.2017.51 (PMC5467240; doi:10.1038/ijo.2017.51)
Supplement: Supplementary Information [file ijo201751x1.docx]

**Appendix**

**Results of sensitivity analyses**

*Exclusion on non-white participants*

About 9% of our sample are of a non-white ethnicity. Re-running multivariable-adjusted models excluding these non-white participants leaves results largely unchanged; decline (95% CI) in physical function among healthy normal-weight participants is -3.69 (-4.20, -3.18) units with worsening relative to this group being -0.26 (-1.16, 0.63) units for unhealthy normal-weight, -0.54 (-1.18, 0.11) units for healthy overweight, -1.08 (-1.80, -0.37) units for unhealthy overweight, -3.47 (-4.94, -2.00) units for healthy obese, and -4.88 (-5.95, -3.82) units for unhealthy obese. Likewise, worsening (95% CI) of bodily pain among healthy normal-weight participants is -0.51 (-1.12, 0.10) units with worsening relative to this group being -0.23 (-1.22, 0.75) units for unhealthy normal-weight, -1.10 (-1.81, -0.40) units for healthy overweight, -1.27 (-2.07, -0.47) units for unhealthy overweight, -2.38 (-4.00, -0.76) units for healthy obese, and -3.63 (-4.81, -2.46) units for unhealthy obese.

For the mobility limitation outcome, ORs (95% CI) as compared with healthy normal-weight are 1.09 (0.84, 1.42) for unhealthy normal-weight, 1.39 (1.16, 1.67) for healthy overweight, 1.61 (1.31, 1.98) for unhealthy overweight, 3.81 (2.51, 5.80) for healthy obese, and 3.94 (2.89, 5.38) for unhealthy obese. For the disability outcome, ORs (95% CI) as compared with healthy normal-weight are 0.70 (0.42, 1.18) for unhealthy normal-weight, 1.57 (1.12, 2.21) for healthy overweight, 2.13 (1.46, 3.09) for unhealthy overweight, 3.56 (1.75, 7.24) for healthy obese, and 8.22 (5.03, 13.41) for unhealthy obese.

*Analysis restricted to participants with at least 2 measures of physical function and bodily pain over follow-up*

The main analyses of decline in physical function and worsening of bodily pain include participants with data on 1 or more out of 8 measures on each outcome. The proportion of participants with just 1 measure of each outcome was small: 1.5% (n=98) for physical function and 1.5% (n=97) for bodily pain. When re-running multivariate-adjusted models including participants with data on at least 2 outcome measurements, the results are essentially unchanged: Decline in physical function and 95% CI among healthy normal-weight participants is -3.68 (-4.20, -3.17) units with decline relative to this group being -0.62 (-1.48, 0.25) units for unhealthy normal-weight, -0.54 (-1.17, 0.09) units for healthy overweight, -1.22 (-1.92, -0.51) units for unhealthy overweight, -3.52 (-4.92, -2.12) units for healthy obese, and -5.03 (-6.07, -3.99) units for unhealthy obese. Likewise, worsening of bodily pain and 95% CI among healthy normal-weight participants is -0.50 (-1.11, 0.11) units with worsening relative to this group being -0.38 (-1.33, 0.57) units for unhealthy normal-weight, -1.12 (-1.82, -0.43) units for healthy overweight, -1.31 (-2.09, -0.53) units for unhealthy overweight, -2.21 (-3.76, -0.67) units for healthy obese, and -4.17 (-5.31, -3.02) units for unhealthy obese.

*Examining sex differences in outcome associations*

In Figure 1, estimates of absolute change in scores for physical function and bodily pain for the main reference group of interest (healthy normal-weight) are computed with men as the reference group. Findings would be very similar if women were used as the reference group for sex given that there was no sex interaction in relation to change in physical function (p-interaction=0.925). There was borderline indication of sex interaction in relation to change in bodily pain (p-interaction=0.054) and we therefore re-ran this analysis stratified by sex, change in bodily pain for men (n=4433) was -0.55 (-1.19, 0.10) units among healthy normal-weight, differences being -0.01 (-1.00, 0.98) units among unhealthy normal-weight, -1.36 (-2.15, -0.56) units among healthy overweight, -1.33 (-2.16, -0.50) units among unhealthy overweight, -2.97 (-5.31, -0.62) units among healthy obese, and -3.46 (-4.85, -2.07) units among unhealthy obese. For women (n=1810), change in bodily pain was 0.11 (-1.44, 1.65) units among healthy normal-weight, differences being -2.82 (-5.56, -0.08) units among unhealthy normal-weight, -0.33 (-1.73, 1.07) units among healthy overweight, -1.08 (-3.09, 0.92) units among unhealthy overweight, -1.73 (-3.95, 0.49) units among healthy obese, and -4.67 (-6.73, -2.61) units among unhealthy obese. Among men the pattern of results is similar as with main sex-combined results, while among women the difference in bodily pain appears largely with unhealthy obesity. It is however unclear how much of this pattern is due to different sample sizes in stratified models (4433 men, 1810 women).

As noted in the main results text, no evidence for sex interaction in relation to mobility limitation or disability was found.

*Differences in participant characteristics between those included and excluded from analyses of mobility limitation and disability*

There was a larger participant drop-out for outcomes of mobility limitation and disability than for continuous outcomes of physical functioning and bodily pain. This was partly due to collection of mobility/disability data starting later than for other outcomes (the first occasion being in 2002/04, nearly 10 years after baseline). Furthermore, fewer participants had data on mobility than for disability, partly due to mobility requiring an objective test of walking speed in a clinic whereas disability only required a questionnaire.

Upon examination, compared with participants who had BMI/metabolic data (the initial prerequisite for inclusion) and also had data on mobility (n=5507), those who had BMI/metabolic data but had missing data on mobility (n=1134) (who did not complete the walking speed test) were on average older (51.10 vs 49.22 years, p<0.001), more likely to be female (34.7% vs 28.22%, p<0.001), more likely to be of a non-white ethnicity (13.32% vs 8.52%, p<0.001), and more likely to be of the lowest occupational position (27.12% vs 13.31%, p<0.001). Those with missing mobility data also had more adverse health behaviours by way of a higher smoking prevalence (21.21% vs 11.82%, p<0.001) and a higher likelihood of consuming fruit and vegetables less than daily (46.47% vs 37.43%, p<0.001), but they were no less likely to consume high amounts of alcohol (14.11% vs 15.74%, p=0.168) or to be less physically active (3.35 vs 3.56 hours/week, p=0.104). Participants with missing mobility data did show a higher prevalence of obesity (12.61% vs 9.42%, p=0.001) and of metabolic risk factor clustering (39.42% vs 32.61%, p<0.001).

This pattern was similar among participants with missing data on disability; compared with participants who had BMI/metabolic data (the initial prerequisite for inclusion) and also had data on disability (n=5616), those who had BMI/metabolic data but had missing data on disability (n=1025) were on average older (51.06 vs 49.26 years, p<0.001), more likely to be female (33.37% vs 28.58%, p=0.002), more likely to be of a non-white ethnicity (15.90% vs 8.14%, p<0.001), and to be of the lowest occupational position (27.92% vs 13.44%, p<0.001). Those with missing disability data also had more adverse health behaviours by way of higher smoking prevalence (21.17% vs 12.03%, p<0.001) and a higher likelihood of consuming fruit and vegetables less than daily (48.78% vs 37.18%, p<0.001), but were no less likely to consume high amounts of alcohol (13.93% vs 15.74%, p=0.143) or to be less physically active (3.40 vs 3.55 hours/week, p=0.294). Participants with missing disability data showed a higher prevalence of obesity (13.27% vs 9.37%, p<0.001) and of metabolic risk factor clustering (40.98% vs 32.46%, p<0.001).

Together, this suggests that participants with missing data on either binary outcome were generally more socioeconomically disadvantaged and less behaviourally and physically healthy than those with complete data; a phenomenon common to analyses of longitudinal data. However, the impact of this healthy participant selection bias is expected to be relatively modest here given the use of repeated measures on all outcomes of interest (8 measures for continuous outcomes, 3 measures for binary outcomes), compared to what would be expected if a more restrictive sample was used for complete case analyses.

Indeed, when doing complete case analyses of mobility limitation and disability (using only data on the last occasion in 2012/13 for each outcome) in standard logistic regression models (sample n=4052), the age-, sex-, and ethnicity- adjusted effect estimates for mobility limitation are smaller in magnitude; these ORs (95% CI) relative to healthy normal-weight adults are 0.84 (0.60, 1.17) among unhealthy normal-weight, 1.29 (1.04, 1.61) among healthy overweight, 1.66 (1.31, 2.09) among unhealthy overweight, 1.83 (1.17, 2.86) among healthy obese, and 3.14 (2.28, 4.32) among unhealthy obese. This is similar for disability, with ORs (95% CI) relative to healthy normal-weight adults being 1.28 (0.99, 1.66) among unhealthy normal-weight, 1.31 (1.09, 1.57) among healthy overweight, 1.52 (1.24, 1.87) among unhealthy overweight, 2.54 (1.70, 3.80) among healthy obese, and 2.86 (2.10, 3.90) among unhealthy obese. This suggests that this bias may result in underestimating, rather than overestimating, real effects.
